# High-throughput field crop phenotyping: current status and challenges

**DOI:** 10.1270/jsbbs.21069

**Published:** 2022-02-17

**Authors:** Seishi Ninomiya

**Affiliations:** 1 Graduate School of Agriculture and Life Sciences, The University of Tokyo, Nishitokyo, Tokyo 188-0002, Japan; 2 Plant Phenomics Research Center, Nanjing Agricultural University, Nanjing, China

**Keywords:** canopy architectural traits, field phenotyping, CNN, image sensors, SfM-MVS, LiDAR, UAS

## Abstract

In contrast to the rapid advances made in plant genotyping, plant phenotyping is considered a bottleneck in plant science. This has promoted high-throughput plant phenotyping (HTP) studies, resulting in an exponential increase in phenotyping-related publications. The development of HTP was originally intended for use as indoor HTP technologies for model plant species under controlled environments. However, this subsequently shifted to HTP for use in crops in fields. Although HTP in fields is much more difficult to conduct due to unstable environmental conditions compared to HTP in controlled environments, recent advances in HTP technology have allowed these difficulties to be overcome, allowing for rapid, efficient, non-destructive, non-invasive, quantitative, repeatable, and objective phenotyping. Recent HTP developments have been accelerated by the advances in data analysis, sensors, and robot technologies, including machine learning, image analysis, three dimensional (3D) reconstruction, image sensors, laser sensors, environmental sensors, and drones, along with high-speed computational resources. This article provides an overview of recent HTP technologies, focusing mainly on canopy-based phenotypes of major crops, such as canopy height, canopy coverage, canopy biomass, and canopy stressed appearance, in addition to crop organ detection and counting in the fields. Current topics in field HTP are also presented, followed by a discussion on the low rates of adoption of HTP in practical breeding programs.

## Introduction

**Fig. 1.**
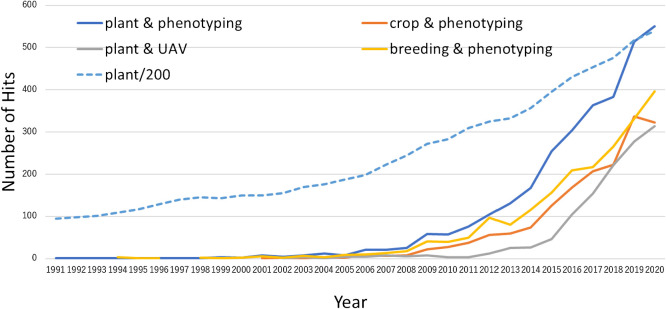
Search results at Web of Science (https://www.webofscience.com/wos/woscc/basic-search) with shown keywords between 1991 and 2020 (access on June 1^st^, 2021). The number of the hits for “plant” was divided by 200 for the comparison of the growth curves.

While plant genotyping has rapidly outperformed Moore’s law of computational power, low-throughput plant phenotyping is seen as a bottleneck of plant science, promoting an intensification of studies on high-throughput phenotyping (HTP) in the last decade. Costa *et al.* (2019) analyzed trends in publications on plant phenomics between 1997 and 2017 and found that the number of these publications increased much more rapidly after 2007 than in other plant science categories. As shown in Fig. 1, this trend accelerated again after 2017. During this period, several plant phenotyping research centers have been founded, including the Australian Plant Phenomics Facility in Australia (APPF) (https://www.plantphenomics.org.au), Jülich Plant Phenotyping Center (JPPC) in Germany (https://www.fz-juelich.de/ibg/ibg-2/EN/Research/ResearchGroups/JPPC/JPPC_node.html), National Plant Phenomics Center in the United Kingdom (NPPC) (https://www.plant-phenomics.ac.uk), Plant Phenotyping and Imaging Research Center in Canada (P2IRC) (https://p2irc.usask.ca), and Plant Phenomics Research Center in Chine (PPRC) (http://pprcen.njau.edu.cn). Alongside these initiatives, a platform for international research collaboration and networking, International Plant Phenotyping Network (IPPN) (https://www.plant-phenotyping.org), was also established.

This boom initially began with the development of indoor HTP technologies for some crops such as maize and soybean as well as model plant species under controlled environments, which subsequently shifted to HTP for crops in fields, also known as field HTP. Although HTP in fields is much more difficult to conduct compared to HTP under controlled environments due to the unstable environmental conditions of the latter, including varying light, shadows, wind, and more complex crop backgrounds, advances in HTP technology have been able to overcome these difficulties, resulting in speedy, efficient, non-destructive, non-invasive, quantitative, repeatable, and objective phenotyping. As a result of these advances, HTP has not only attained the capacity to replace human visual judgments in a much faster and more objective manner, but is also able to evaluate new traits, such as a comparison of time series canopy growth curves (Guo *et al.* 2017) among thousands of genotypes.

Recent advances in HTP have been supported by advances in data analysis, sensors, and robot technologies (Roitsch *et al.* 2019). Machine learning approaches represented by convolutional neural networks (CNN) (Jiang and Li 2020) have also contributed to advances in newly emerged image-analyzing technologies, such as 3D reconstruction by SfM-MVS (structure from motion and multi-view stereo) (Guo *et al.* 2021), which reconstruct 3D structures of objects based on stereo photogrammetry using multiple images of the target objects. Sensor hardware and computer resources have markedly improved, and prices have decreased, making them more popular. The resolution of commercial RGB cameras now stands at 100 million pixels. Similarly, multispectral cameras and light detection and ranging (LiDAR) systems, which are extremely expensive, are now also available at reasonable prices (Guo *et al.* 2021). LiDAR (Guo *et al.* 2021) allows for distance scanning to reconstruct the 3D structures of objects by detecting the distances to the target objects. Even the price of hyperspectral cameras, which exceeded 100,000 USD some years ago, is falling rapidly (Guo *et al.* 2021).

In addition, advances in sensor platforms within the field of robotics have supported the progress of HTP (Zhao *et al.* 2019). Particularly, the recent contribution of advances in unmanned aircraft systems (UASs), also often called unmanned aerial vehicles (UAVs), has been outstanding for HTP, along with several types of UAS-mountable image sensors, such as RGB, multispectral, hyperspectral, and thermal cameras (Guo *et al.* 2021). Similarly, advances in IoT environment sensors, such as Field Server (Hirafuji *et al.* 2013), have been also supported HTP, particularly when considering the importance of understanding G×E (genotype and environment interaction).

This article provides an overview of recently developed HTP technologies, focusing on the canopy-based architectural phenotypes of major crops, such as canopy height, canopy coverage, canopy biomass, canopy stressed appearance, and canopy level crop organ detection and counting. This article does not discuss root phenotyping, which is as important as above-ground phenotyping (Atkinson *et al.* 2019, Uga 2021), since it is reviewed in the same issue (Teramoto and Uga 2022). Instead, current topics in field HTP are discussed, including the challenges associated with promoting the use of machine learning approaches in HTP.

The dynamic and rapid advances being made in HTP has lead stakeholders to expect breeders to adopt HTP in their breeding programs (Fasoula *et al.* 2020, Rebetzke *et al.* 2019, Watt *et al.* 2020). However, the adoption of HTP in practical breeding programs is stagnant (Awada *et al.* 2018, Deery and Jones 2021). In the final part of this review, we briefly discuss the reasons for the low rate of adoption of this technology.

## Canopy height, canopy coverage, and biomass

The estimation of biomass-related traits has been widely studied in satellite remote sensing (Liu *et al.* 2019a). However, considering the current resolution of satellite images, satellite-based biomass estimation models cannot be applied to the average scale of breeding plots. However, UAS-based monitoring is currently the best fit for the scale of the plots. Moreover, the comparatively easy usability and the reasonable cost of UAS promote its use in plant breeding (Guo *et al.* 2021).

### Canopy height

The efficiency of canopy height estimation, which used to be highly laborious, has been dramatically improved by two types of 3D reconstruction technologies: SfM-MVS and LiDAR. SfM-MVS is mainly used with UAS-based RGB (UAS RGB) and/or UAS-based multispectral (UAS multispectral) images, whereas LiDAR systems are usually either fixed obliquely looking down fields or mounted on mobile platforms, such as vehicles and gantries. Currently, the 3D reconstruction of canopies using SfM-MVS with UAS images is more scale-efficient than that using ground-based LiDAR. However, 3D reconstructions by SfM-MVS at times fail, depending on the quality of the acquired images and the complexity of the canopy structures. This method also requires more computational resources than LiDAR. Considering that reasonably priced UAS-mountable LiDAR systems are becoming increasingly available, we expect LiDAR to take the lead in 3D reconstruction in the near future (Guo *et al.* 2021).

Examples of canopy height estimation by SfM-MVS have been provided for wheat (Cai *et al.* 2018, Hassan *et al.* 2019, Khan *et al.* 2018,Yue *et al.* 2018b), barley (Wilke *et al.* 2019), rice (Kawamura *et al.* 2020), maize (Wang *et al.* 2019, Ziliani *et al.* 2018) and sorghum (Hu *et al.* 2018, Watanabe *et al.* 2017), while examples of canopy height estimation by LiDAR have been provided for wheat (Friedli *et al.* 2016, Jimenez-Berni *et al.* 2018, Walter *et al.* 2019a, 2019b), rice (Phan *et al.* 2016, Tilly *et al.* 2014), corn (Friedli *et al.* 2016), soybean (Friedli *et al.* 2016), cotton (Sun *et al.* 2018), and peanut (Yuan *et al.* 2019). Hu *et al.* (2018) proposed a method to calibrate the estimated values by using small number of manually observed values. Note that the estimation of canopy heights from 3D point clouds constructed by SfM-MVS or LiDAR differ among these studies.

### Canopy coverage, senescence, and seedling emergence

Canopy coverage is a good indicator of crop growth, particularly when it is obtained sequentially to obtain a growth curve. While it was almost impossible to obtain this type of curve easily, high-throughput imaging by UASs or ground vehicles has made this a reality.

Image-based canopy coverage estimation requires accurate crop segmentation from the background. Historically, simple thresholding based on a value determined by maximum likelihood classification or color indices, such as ExG (Woebbecke *et al.* 1995), have been used for such segmentations. Guo *et al.* (2013) raised questions about the robustness of existing methods under varying illumination with heavily shadowed patches of outdoor fields, and proposed a machine learning based segmentation method, DTSM (decision tree segmentation model), the accuracy and the robustness of which have been confirmed in wheat, rice, cotton, sugarcane, and sorghum (Duan *et al.* 2017, Guo *et al.* 2013, 2017), and which is now widely used as a published application, EasyPCC (Guo *et al.* 2017), in plant science. The canopy coverages of wheat (Jimenez-Berni *et al.* 2018) and cotton (Sun *et al.* 2018) have also been estimated using ground-based LiDAR observations. Similarly, the senescence or stay-green of wheat, maize, and sorghum has been evaluated by UAS RGB or multispectral images (Hassan *et al.* 2018, Liedtke *et al.* 2020, Makanza *et al.* 2018). Using UAS-RGB images, the emergence of wheat, rice, maize, and potato was evaluated (Li *et al.* 2019, Liu *et al.* 2017, Velumani *et al.* 2021, Wu *et al.* 2019). In a unique study, Bruce *et al.* (2021) assessed the variation of soybean pubescence using UAS multispectral images.

### Biomass and LAI

Unlike the majority of height and canopy coverage estimations, the estimations of aboveground biomass (AGB) and leaf area index (LAI) usually require some regression to estimate the target trait values. There are two types of estimation. The first type uses vegetation indices, such as normalized difference vegetation index (NDVI), calculated based on spectral reflectance values from multispectral or hyperspectral images captured by UAS cameras or ground cameras, while the second type uses the architectural values of plants, such as the height and volume of plants obtained from 3D reconstruction data. The AGB and LAI estimations of wheat (Hu *et al.* 2021, Khan *et al.* 2018, Lu *et al.* 2019, Yao *et al.* 2017, Yue *et al.* 2018a) and rice (Shu *et al.* 2021, Tanger *et al.* 2017, Wang *et al.* 2021c) are examples of the first type, while estimations of wheat (Deery *et al.* 2020, Jimenez-Berni *et al.* 2018, Walter *et al.* 2019b), soybean (Herrero-Huerta *et al.* 2020), and cotton (Sun *et al.* 2018) are examples of the second type. There are also examples where both types are mixed, such as rice (Jiang *et al.* 2019) and corn (Michez *et al.* 2018). Riera *et al.* (2021) used a completely different approach to estimate soybean yield, choosing to count the number of pods from images captured by a ground robot cart.

### Crop stress assessments

Methods for the high-throughput phenotyping of abiotic and biotic stresses on crops, including drought, pests, and diseases, have also advanced rapidly, making use of advances in machine learning technologies (Singh *et al.* 2016, 2018, 2021). The scope of these works vary from the leaf-scale level to the field level.

### Disease assessments

CNN has played an important role in the identification of biotic stress, particularly at the leaf or individual plant level (Boulent *et al.* 2019). For example, nine different stress-induced phenotypes in soybean leaves (four different diseases, two nutritious deficiencies, herbicide injury, sudden death syndrome, and normal) of soybean single leaves were highly accurately classified and quantified (Ghosal *et al.* 2018) using CNN, and ten different stressed appearances on tomato leaves (gray mold, canker, leaf mold, plague, leaf miner, whitefly, low temperature, nutritional excess or deficiency, powdery mildew) were accurately classified using CNN (Fuentes *et al.* 2017, 2018). Furthermore, an accurate and qualitative assessment of disease at the leaf level can help in the identification of efficient resistant genes, as was done for *Septoria tritici* blotch (STB) in wheat (Yates *et al.* 2019). Technologies that utilize intact leaf images taken under natural conditions have also seen advances for use in the accurate recognition of diseases (Fuentes *et al.* 2017, 2018, Johnson *et al.* 2021).

While studies at the leaf-level could be used to replace observations by experts and provide objective and repeatable evaluations, improvements in assessment efficiency when applied in the field have yet to be achieved. Thus, canopy-level stress high-throughput phenotyping, mainly by UASs, has also been studied, which is expected to see a dramatic acceleration of its application in the assessment of stress in plant breeding (Barbedo 2019). Following the success of disease assessment using ground mobile platforms, including for sugar beet cercospora leaf spot (Atoum *et al.* 2016) and wheat STB (Walter *et al.* 2019a), field level disease assessment by UAS has been widely performed using RGB and/or multispectral images with CNN (Guo *et al.* 2021): northern corn leaf blights (DeChant *et al.* 2017, Wiesner-Hanks *et al.* 2019), wheat yellow rust (Su *et al.* 2018), wheat stripe rust (Schirrmann *et al.* 2021), rice sheath blight (Zhang *et al.* 2018), potato late blight (Duarte-Carvajalino *et al.* 2018, Sugiura *et al.* 2016), soybean foliar diseases (Tetila *et al.* 2017), sugar beet cospora leaf spot (Altas *et al.* 2018, Jay *et al.* 2020), peanut tomato spot wilt (Patrick *et al.* 2017), radish Fusarium wilt (Dang *et al.* 2020, Ha *et al.* 2017), and soybean iron-deficient chlorosis (Dobbels and Lorenz 2019). Taking into account the falling prices of hyperspectral cameras, we can expect this technology to be widely applied for disease assessment in the coming years. Thomas *et al.* (2018) used this technology for barley powdery mildew at a ground-based phenotyping facility, while Joalland *et al.* (2018) used the same technology to assess tolerance to sugar beet cyst nematode (SBCN).

### Water stress

Canopy surface temperature (CT) is a good indicator of stomatal conductance (Moller *et al.* 2007, Seguin *et al.* 1991) because plant surfaces are cooled in proportion to the evaporation rate. Recently, several different types of thermal cameras mounted on UAS have become commercially available (Guo *et al.* 2021), and their use in monitoring CT has been confirmed (Deery *et al.* 2019, Sagan *et al.* 2019).

The CT is constantly and rapidly changing according to the environmental conditions, including light, temperature, and wind. As a result, consistent and repeatable measurements over crop canopies are difficult (Perich *et al.* 2020). However, several ideas have been proposed by researchers to achieve reliable CT measurements for maize (Zhang *et al.* 2019), fruit trees (Han *et al.* 2021), wheat (Perich *et al.* 2020), soybean (Crusiol *et al.* 2020), and barley (Hoffmann *et al.* 2016). For example, Perich *et al.* (2020) used the heritability of CT to identify the optimal timing of the measurement.

Structural changes in plants, such as leaf wilting, which is detectable by image analysis, can also be an indicator of water stress (Srivastava *et al.* 2017, Wakamori and Mineno 2019). Another way to estimate water stress is to use models or indices based on hyperspectral or multispectral images (Asaari *et al.* 2019, Romero *et al.* 2017, 2018, Thorp *et al.* 2018). Flooding stress on soybeans has also been previously assessed using UAS multispectral and thermal images (Zhou *et al.* 2021).

### Salinity stress

Salinity stress usually causes growth deficiencies. As a result, phenotyping methods for biomass-related traits can be used to identify salinity stress by comparing with control plants. This method was used by Johansen *et al.* (2019), who evaluated the response of wild tomato genotypes to salinity stress by comparing growth curves based on canopy coverage estimated from the UAS RGB and multispectral time-series images. Similarly, Ivushkin *et al.* (2019) showed that the hyperspectral physiological reflectance index (PRI, Gamon *et al.* 1992) obtained from hyperspectral images could be used to identify the stress of treated quinoa plants compared to control plants.

### Lodging

UAS canopy monitoring provides an opportunity for the high-throughput and quantitative measurement of canopies to evaluate the extent of lodging. The canopy height estimation methods based on SfM-MVS or LiDAR can be directly used for lodging assessments (Singh *et al.* 2019, Wilke *et al.* 2019), whereas lodging assessment based on image features, canopy coverage, and NDIV from UAS multispectral images (Han *et al.* 2018, Sun *et al.* 2019) or a combination of selected bands of hyperspectral images (Wang *et al.* 2021c) have also been proposed.

### Weed identification

Although weed detection using ground vehicles is well documented, particularly for localized precision herbicide applications, few studies have reported on UAS-based weed detection (Singh *et al.* 2020). UAS-based weed detection is particularly important when crop traits, such as biomass and canopy coverage, are estimated from fields contaminated by weeds. De Castro *et al.* (2018) proposed a method to segment weeds in sunflower and cotton fields using random forest classification based on features derived from UAS RGB and multispectral images and crop height estimated from UAS RGB images. This study aimed to identify broad-leaf weeds and grass weeds (Torres-Sánchez *et al.* 2021). Huang *et al.* (2018) demonstrated that rice and weeds can be classified based on UAS RGB images using a CNN model, fully convolutional network (FCN) and transfer learning (Jiang and Li 2020). While the current methods are not applicable to complex fields where weeds of various species are intermingled, Skovsen *et al.* (2021) demonstrated that CNN models can classify white clover, red clover and weed from rather complicated canopy images, using synthetic training data which is discussed in the later part of this paper. Variations in hyperspectral reflectance among certain weeds and crops have been reported (Singh *et al.* 2020), indicating that UAS hyperspectral images can be used to segment weeds from crops.

## Canopy-level crop organ detection and counting

The development of automatic crop organ detection and counting technologies in outdoor fields has been a newly emerging area in the last 5 years, occurring alongside advances in image analyzing technologies, mainly based on machine learning. Crop organ detection and counting in fields is hindered by the variations in the environmental conditions, such as light, shadows, wind, rain, and heavy occlusion of the organs, in contrast to controlled indoor conditions. In breeding fields, the intraspecific variations in shape, size, and color among different genotypes accelerate these difficulties. Despite such difficulties, recent studies on crop organ detection and counting have reported great success, as exemplified below.

### Rice panicle detection and counting

A pioneering study (Guo *et al.* 2015) performed an accurate automatic detection of rice flowering panicles based on time series RGB images captured by ground-based cameras, using the scale-invariant feature transform (SIFT) (Lowe 2004), bag of visual words (BoVWs) (Csurka *et al.* 2004), and a support vector machine (SVM). The study showed that, visually, very small events, such as rice flowering (anthesis), which occurs at particular times on particular days on particular parts of the panicles, could be automatically detected from images taken under varying natural conditions. Similarly, Desai *et al.* (2019) used a CNN model, ResNet-50 (Jiang and Li 2020), to detect rice flowering panicles instead of image feature extractions, such as SIFT, and showed that the heading date of the rice canopy could be estimated using the daily cumulative distribution of the detected number of flowering panicles.

Methods to automatically detect and count rice panicles in paddy rice canopies were proposed using CNN (Lyu *et al.* 2021, Xiong *et al.* 2017, Zhou *et al.* 2019). Lyu *et al.* (2021) used UAS RGB images captured at comparatively low altitudes (1.2 m) with a CNN model, Mask R-CNN (Jiang and Li 2020), and achieved a counting precision of 0.82 (*precision* = *T_p_*/*T_p_* + *F_p_* while *recall* = *T_p_*/*T_p_* + *F_p_*, where *T_p_*, *F_p_*, and *F_p_* are the numbers of true-positive, false-positive, and false-negative in the detections, respectively). The panicle annotation dataset (38,799 patches) used by Lyu *et al.* (2021) was expanded to 50,730 by filtering the results of the automatic detection of panicles (Wang *et al.* 2021b).

### Wheat spike detection and counting

The detection and counting of wheat spikes has been widely performed using CNN models, challenging several of the difficulties experienced under natural conditions (Alkhudaydi *et al.* 2019, Fernandez-Gallego *et al.* 2018, Hasan *et al.* 2018, Madec *et al.* 2019, Sadeghi-Tehran *et al.* 2019, Xiong *et al.* 2019, Zhao *et al.* 2021a).

Sadeghi-Tehran *et al.* (2019) proposed DeepCount to detect and count wheat spikes from ground-based RGB images by combining an image segmentation method, SLIC (Achanta *et al.* 2012), and a CNN model, VGG (Jiang and Li 2020), while Madec *et al.* (2019) used a CNN model, Faster-R-CNN (Jiang and Li 2020), to detect and count wheat spikes based on ground-based high-resolution RGB images to estimate the ear density, achieving *R*^2^ = 0.91 in spike counting. Hasan *et al.* (2018) also used Faster R-CNN, achieving *R*^2^ = 0.93 in spike counting regardless of spike growth stage with RGB images captured from a hand-pushed cart. Xiong *et al.* (2019) developed a large annotation dataset of wheat spikes and developed a CNN model, TasselNetv2, to count wheat spikes and improve the structure of TasselNet (Lu *et al.* 2017). TasselNetv2 achieved not only good spike counting accuracy, even for lower resolution ground-based RGB images than those used by Madec *et al.* (2019), but also a faster performance than TasselNet. Lu and Cao (2020) proposed TasselNetV2+, adding several modifications to the algorithm of TasselNetV2 to improve the computational efficiency of wheat spike detection and counting while retaining the accuracy.

Using the UAS-RGB images captured at altitudes between 7 and 15 m, Zhao *et al.* (2021a) achieved a wheat spike detection accuracy (*IoU*) of 0.94 using a CNN model, YOLOv5 (Jiang and Li 2020). In another study, Zhao *et al.* (2021b) proposed a method for automatically determining the heading date of wheat spikes. Instead of directly detecting the emergence of spikes, they used the inflection points of the canopy growth curves estimated from UAS RGB images as an indicator of heading. The mean absolute error of the estimated heading date was 2.81 days. Jin *et al.* (2019) estimated the stem density of wheat using RGB images of stem cross-sections left on the ground after the harvest using Faster R-CNN, and found that the value was a good proxy of ear density.

David *et al.* (2020, 2021) provided a large-scale open benchmark dataset of wheat images through a multilateral international collaboration. The dataset created in 2020 (David *et al.* 2020) included 4,700 high-resolution wheat images of various genotypes and various growth stages collected from several countries around the world and 190,000 wheat spike annotations, to accelerate the development of spike detection algorithms. The dataset was used at a global competition, Global Wheat Head Detection (https://www.kaggle.com/c/global-wheat-detection), in which 2,245 teams from around the world participated. The dataset was updated by adding 1722 images from 5 additional countries with 81,553 additional wheat heads (David *et al.* 2021) where the dataset was reexamined and relabeled to improve the dataset quality.

### Other cereal crops

Lu *et al.* (2017) developed a CNN model, TasselNet, to count maize tassels using ground-based RGB images captured in outdoor fields, whereas Mirnezami *et al.* (2021) developed a method to detect maize tassels and track the development of each individual tassel regardless of the shape variations among several genotypes, combining several models, such as a CNN model, RetinaNet (Jiang and Li 2020), based on time-series ground-based RGB images. Guo *et al.* (2018) developed a sorghum head detection and counting algorithm from UAS RGB images captured at an altitude of 20 m. They used a machine learning-based plant segmentation algorithm, DTSM (Guo *et al.* 2013), to detect sorghum heads of various colors. Because some of the regions detected as sorghum heads contained more than one head, they estimated the number of heads in each of the detected head regions by SVM with the eleven image features, such as area, perimeter, and roundness of the regions, and achieved a precision/recall of 0.87/0.98 for the detection and *R*^2^ = 0.84 for head counting. TasselNetV2+ (Lu and Cao 2020) achieved improved computational efficiency also for maize tassel and sorghum head detections.

### Fruit detection and counting

A CNN model named Deepfruits (Sa *et al.* 2016) based on Faster R-CNN was one of the first studies to demonstrate the power of CNN for fruit detection. Deepfruit employed both RGB and NIR images as multimodal inputs and was successfully applied to fruits of seven different crop species: sweet pepper, melon, apple, avocado, mango, orange, and strawberry. Kang and Chen (2020) proposed a CNN model, LedNet, to detect apples in orchards, achieving an accuracy (*IoU*) of 0.85. To promote fruit detection studies, Häni *et al.* (2020) published a benchmark dataset for apple detection and segmentation that contained 1,000 images and 41,000 annotated instances of apples.

Mu *et al.* (2020) succeeded in detecting highly occluded immature green tomatoes using CNN models (R-CNN and ResNet-101, Jiang and Li 2020), achieving *R*^2^ = 0.87. Yeom *et al.* (2018) estimated the number of open cotton balls using image feature extraction on the UAS RGB images at an altitude of 15 m. Riera *et al.* (2021) estimated the number of soybean pods in each breeding plot as the basis for yield estimation using CNN models (VGG and RetinaNet), wherein the images were captured using a video camera mounted on a small field robot that moved between the rows of the plot.

## New challenges in high-throughput field phenotyping

### Model-assisted phenotyping

Model-assisted phenotyping is an approach used to estimate phenotypes that cannot be directly observed using crop models parameterized by observable phenotypes. Simple examples have already been introduced in the biomass estimation section of this article. Occlusion is an unavoidable issue when phenotyping canopy structures, particularly in the late growth stage, when the foliage architecture becomes complex. For example, one study found that the accuracy of the total leaf area and leaf number of soybean plants estimated from UAS images was much worse in the late growing stage than in the early growing stage (Liu *et al.* 2021a). To overcome this issue, Liu *et al.* (2019b) proposed a modeling workflow called the digital plant phenotyping platform (D3P) for wheat, coupling an L-system-based wheat architectural model (ADEL-wheat, Fournier *et al.* 2003) and observations by HTP. They conducted a simulation study to estimate the model parameters and a green area index (GAI, green plant area per ground area) by the assimilation data from the green fraction estimated from RGB images of the canopy to D3P. As a result, they demonstrated that some architectural parameters, such as phyllochron, lamina length of the first leaf, rate of elongation of leaf lamina, number of green leaves at the start of leaf senescence, and minimum number of green leaves, and GAI were accurately estimated. Data assimilation, in which model parameters are dynamically updated using observed data, is commonly used in satellite-based crop monitoring studies, such as yield estimation (Zhang *et al.* 2016).

Similar data assimilation has been used in several studies, such as Blancon *et al.* (2019), Roth *et al.* (2020), and Peng *et al.* (2021), to estimate directly unobservable trait values. Blancon *et al.* (2019) estimated the parameters of a green leaf area index (GLAI) dynamic model of maize using the estimated GLAI from the empirical relationship between multispectral reflectance obtained from UAS multispectral images at an altitude of 60 m and GLAI manually measured at the ground level. They found that the GLAI dynamic was accurately estimated (*R*^2^ = 0.9), as well as the model parameters, including the maximum leaf area and leaf longevity. Additionally, they found that the model parameters and GLAI dynamics were highly heritable (0.65 ≤ *H*^2^ ≤ 0.98). Similarly, Roth *et al.* (2020) estimated the beginning of stem elongation, the rate of plant emergence, and the number of tillers of wheat seedlings by SVM and crop modelling based on timeseries multi-view angle UAS RGB images at an altitude of 18 m, achieving a tiller number estimation accuracy of *R*^2^ = 0.86.

### Latent space phenotyping

Ubbens *et al.* (2020) proposed the latent space phenotype (LSP) to evaluate time-course phenotypic changes caused by abiotic stress factors, such as drought, nitrogen deficiency, and salinity. These phenotypic changes can be very complicated and depend on many factors. As such, it is not easy to quantify the changes, and humans are not always able to easily identify the different phenotypic responses to different treatments. The authors first obtained abstract low-dimensional vectors that discriminate between time-series images captured under stressed and control conditions by encoding the original images using CNN and an extension to recurrent neural network (RNN) (Jiang and Li 2020) and long short-term memory (LSTM) (Jiang and Li 2020). The encoding process was not different from the widely used CNN-based phenotypic discrimination, such as disease identification (Singh *et al.* 2016, 2018, 2021). However, Ubbens *et al.* (2020) added a decoding process to recover low-dimensional vectors from the original images by CNN training. The outputs of the decoding process represented the image expressions of the different responses to treatment. They defined the distance between two decoded images and used the sum of the distances from the first image to the last image of the decoded time-series images as the LSP, which represents the difference in time-course responses to the treatments. Then, they demonstrated some use cases of LSPSs. For example, a QTL analysis based on the LSPs obtained from the C4 model plant, Setaria RILs, subjected to water stress treatments, was used to identify the same QTLs related to water stress, as reported by Feldman *et al.* (2018).

Gage *et al.* (2019) also used the concept of LSP for the point cloud data acquired by a LiDAR mounted on a phenotyping rover in maize fields to evaluate variations in plant architectures among 698 hybrid genotypes, as 3D point cloud data cannot be directly parameterized to understand variation. First, they created a 2D marginal frequency distribution of the 3D point cloud of maize crops in each plot. Then, they used two methods of dimension reduction to map the original 2D distribution to LSPs: an autoencoder and principal component analysis (PCA). They trained the CNN encoder and decoder so that the original 2D distribution images (input) were encoded to 16-dimensional vectors as LSPs, and the vectors were decoded back to 2D distribution images (output), minimizing the loss based on the mean square error between the input and the output. They also used PCA to obtain 16 principal component scores as the LPSs. Some of the LSPs showed high heritability as manually measured architectural traits. In other words, extremely complicated 3D point clouds were summarized to a few latent variables using either a CNN autoencoder or PCA on 2D frequency distributions of the 3D point clouds, and the latent variables were linked to heritability. Their results also showed that the partial least squares (PLS) regression model based on the LSPs was able to predict some of the manually measured traits well.

One possible way to understand the relationship between the latent variables and observable phenotypes is to intentionally fluctuate the latent variables and decode them back to images to see how the fluctuation changes the images. A similar approach of dimension reduction from images and image recovery was successful in previous simpler image analysis studies on plant phenotyping, such as Yoshioka *et al.* (2004) and Furuta *et al.* (1995). We also expect the concept of LSP to be readily applied to hyperspectral images where a tremendously large number of dimensions need to be handled, and in which it is difficult to intuitively infer the data structure.

### Leaf segmentation and reconstruction in canopy

Leaves and roots are important organs for maintaining photosynthesis. Although leaf canopies have historically been evaluated as a mass of leaves, the automatic segmentation of individual leaves has been recently challenged in crops, such as sugar beet (Xiao *et al.* 2020), barley (Paulus *et al.* 2014), maize (Miao *et al.* 2020), and wheat (Srivastava *et al.* 2017), based on 3D-point clouds constructed by SfM-MVS or LiDAR. Once such organ segmentations are successful from the point clouds, surface reconstruction of the segmented point clouds for each organ becomes necessary, as described by Ando *et al.* (2021). However, these studies focused on the individual plant level and cannot be directly linked to canopy performance, such as light interception efficiency, in the field. Understanding the leaf canopy foliage structure in a crop population is directly linked to the evaluation of photosynthesis through the ability to intercept light and the productivity of the canopy, expecting to identify genes in the architectural structure.

Leaves are often heavily occluded in the crop canopy. As shown by Isokane *et al.* (2018), the detailed 3D architectural structure of an individual plant can be reconstructed using CNN and multiview images, even if some parts of the plant are not visible from the outside. This highlights the possibility of using virtual crop populations constructed based on the detailed 3D architectural information acquired at the individual plant level for the comparison of photosynthetic performances among the virtual canopies with different plant architectures, as attempted by Liu *et al.* (2021b).

### Interoperable data integration and data management platform

Alongside the rapid advances of HTP, the amount of data accumulated, including image data, is enormous. Building data management platforms for phenotypic data, as well as other omics data and environmental data, is tremendously important for plant science research, in combination with the development of data analysis technologies (Coppens *et al.* 2017). Because most of the data ever accumulated are managed in a proprietary format within a research organization, or even by a person who generates the data, data sharing among different organizations is rather inefficient. To accelerate collaborative research and realize interoperability, it is strongly recommended to integrate various types of data generated by different organizations.

To accelerate such interoperable data management and the development of data platforms, several international standards, such as Crop Ontology (Shrestha *et al.* 2012), which defines the relationships among crop-related vocabularies, MIAPPE (Minimum Information About a Plant Phenotyping Experiment) (Ćwiek-Kupczyńska *et al.* 2016), which proposes metadata standards for the data related to plant phenotyping, and BrAPI (Breeding API) (Selby *et al.* 2019), which efficiently bridges the breeding-related data and software developments, have been proposed. Utilizing these international standards, GnpIS, a data repository for plant phenomics, was developed (Pommier *et al.* 2019). This repository allows for long-term access to datasets according to the FAIR principles (Findable, Accessible, Interoperable, and Reusable) (Wilkinson *et al.* 2016), covering phenotypic and environmental data, and ensures interoperable data integration between phenotypic and genotypic datasets. The use of GnpIS also guarantees interoperability with other data repositories by using international standards that enable such data links.

Many phenotyping studies using machine learning have published training data on paper publications. We have learned that compiling image data sets of wheat spike detection from several organizations around the world has accelerated related studies (David *et al.* 2020) and expect this activity should move across species aiming at a similar image archive as ImageNet (https://image-net.org/index.php) (Russakovsky *et al.* 2015). This archive has been fundamental in supporting the rapid development of general object recognition. As mentioned several times in this paper, 3D reconstruction technologies have been widely used in plant phenotyping to generate 3D information. Griffiths (2020) proposed a 3D print repository for plant data with data standardization, discussing the future perspective of 3D printing technologies in plant phenomics.

## Easing training data provisions in machine learning approaches

As mentioned above, image analyses with machine learning technologies, including CNN, have been successfully applied to plant phenotyping, replacing human visual assessments with even higher accuracy. However, the machine learning approach requires the provision of training datasets. In general, the development of training datasets requires human visual annotations to manually label target objects, costing both labor and time. Moreover, a machine learning-based model developed in a domain cannot be applied to other domains. To ease such annotation costs, several solutions have also been proposed in plant phenomics.

### Acceleration of annotation process

Ghosal *et al.* (2019) proposed a weakly supervised deep learning approach inspired by active learning for the detection and counting of sorghum heads in UAS RGB images using CNN models (RetinaNet and ResNet-50). In the weakly supervised approach, a CNN model was first trained with a small number of images. Then, false negatives and false positives generated during the validation process of the model were added to the original training data set repeatedly until a good detection performance is achieved. These authors showed that a model trained with 40 images by the weakly supervised approach achieved the same detection performance (*R*^2^ = 0.88) as a model trained with 283 images. Although the proposed method still requires human interaction to identify false-negative and false-positive results after the validation process, the annotation time was roughly four times faster on average. Usually, the annotation process requires labeling objects by drawing bounding boxes around objects, and the process performed visually by humans is time-consuming. To simplify this process, Chandra *et al.* (2020) proposed a point supervision approach, where the first step of the annotation was performed by clicking the inside of each object instead of drawing bounding boxes, followed by the automatic proposals of object regions for the next cycle of the weakly supervised training, resulting in a significant reduction in the annotation time.

### Domain adaptation

A machine learning-based model, such as a CNN model trained in a particular domain, cannot be usually applied in another domain. For example, an orange fruit detection model from orange trees, which is supervised by the manual annotation of orange fruits, may not perform well or may even be totally useless in apple fruit detection. Therefore, a new training process based on images obtained from apple fruits is usually required. This approach is rather ad hoc, requiring the building of domain-specific models unlimitedly. In this context, expanding the coverage of a model trained in a domain to another domain without providing the training data set for the new domain, called domain adaptation, has been a hot topic in machine learning studies. Zhang *et al.* (2021) proposed a domain adaptation method for fruit detection using a CNN model, CycleGAN (Zhu *et al.* 2017), based on GAN (Generative Adversarial Networks) (Goodfellow *et al.* 2014). CycleGAN is often used to transform images in a domain to those in another domain to learn the relationship between the two domains. Zhang *et al.* (2021) applied this feature of CycleGAN to automatically transform the training images manually annotated for orange fruit detection to the training images for fruits of other crops, such as apple and tomato fruits, without conducting the annotation process for those new crops. They trained a CycleGAN model to transform single orange images into single apple images, and the orange images of orange trees taken in an orchard were transformed into fake apple images using the trained CycleGAN. The fake images were used to train a CNN model, the Improved-Yolov3 (Jiang and Li 2020) model, to detect apples using the annotation information made on the original orange tree images, such as locations and bounding-box sizes, as pseudo-labels. The proposed method also included filtering out improper pseudo-labels to increase the accuracy of the detection. The results showed that the precision and recall of the detections by the models trained based on the pseudo-labels were as high as 0.89/0.92 and 0.91/0.94 for apples and tomatoes, respectively.

### Data augmentation and synthetic data

Image data augmentation is comparatively a simple idea to stretch the scale of training data using existing training images. This stretch is expected to improve the robustness of the trained model preventing overfitting without additional costs for time-consuming processes, such as manual annotation. The simplest data augmentations are geometric transformations, such as flipping, rotation, cropping, shifting, zooming, and noise injection, randomly given to the original training images to increase the volume of training data. Color space transformation on the original training images is another example of augmentation. In addition to widely used image augmentations, the concept of synthetic data, sometimes called domain randomization, has been applied to plant phenomics to unload the annotation process and construct even more robust models. For example, (https://arxiv.org/abs/1807.10931) successfully trained a leaf instance segmentation model based on Mask R-CNN for *Arabidopsis* by combining existing real training images with artificially generated images from a 3D rendering model. Shete *et al.* (2020) developed TasselGAN, which could synthesize maize tassel images to be used as training data for tassel detection and segmentation, by merging artificial tassel images and sky images generated.

Toda *et al.* (2020) demonstrated a successful case of artificial data synthesis in their segmentation of crop seeds. First, they provided 20 single-seed images of 20 barley cultivars and manually annotated a bounding box for each of the seed images. Then, they repeated the process to locate randomly selected single seed images on a background with random rotations, allowing for a certain level of seed overlap to ensure that an image of the seed pool of a genotype was synthesized. Then, they generated 1,200 similar seed pool images and trained Mask R-CNN for the segmentation of barely seeds in barley seed pool images, where some of the seeds were overlapped and occluded, achieving very good segmentation performance against real-world seed pool images. They also showed that the segmented seed images were useful for seed morphological characterization, and that the proposed method was generally applicable to seed segmentations of other crops, such as wheat, rice, oat, and lettuce.

### Understanding CNN black boxes

While CNN has shown great success in plant phenotyping, sometimes overperforming human visual judgment, they were left as black boxes in many of the cases. Understanding black boxes sometimes provides useful knowledge. For example, Ghosal *et al.* (2018) built a CNN model to accurately classify several leaf diseases in soybean and identify a key layer for classification. The heatmap pattern of the key layer was then used for the quantification (grading) of the diseases. Toda and Okura (2019) attempted to understand the inside functions of the black boxes of CNN disease classifiers trained with publicly available plant disease images by visualizing the status of neurons and layers. As a result, they discovered that CNN identified the disease in a manner similar to human visual judgment. With these findings, they demonstrated that some of the layers that did not contribute to the classifications could be eliminated without degrading the classification performance.

## Discussion

This paper provides a summary of the current status and challenges of HTP, focusing mainly on the technologies used in outdoor fields for architectural crop traits, leaving the topic of root phenotyping uncovered. Based on our findings, we expect HTP to replace methods that are tedious, low-throughput, subjective, destructive, invasive, subjective, and qualitative, by covering a broader breeding field in a shorter time, thereby contributing to more efficient plant breeding.

Some studies, such as Tanger *et al.* (2017) and Walter *et al.* (2019a), have discussed the usability of HTP in practical breeding. Tanger *et al.* (2017) compared the usability of HTP in rice breeding, targeting a new mapping population of over 1,500 RILs. They were able to scan over 4,500 plots of a 1.5 ha experimental field within two hours using a boom-sprayer-based ground vehicle with multispectral reflectance sensors, ultrasonics canopy height sensors, and infrared sensors. As a result, they estimated the vegetation index and height, and discovered that the QTLs identified for the traits obtained by HTP, even during the flowering stage, corresponded to the QTLs of the manually observed yield-related traits. They concluded that HTP could accelerate breeding, allowing researchers to estimate the breeding values and the effect of QTLs at a much earlier stage, in addition to very efficient data collection. Walter *et al.* (2019b) estimated the biomass and canopy height of wheat breeding fields using LiDAR mounted on a ground vehicle, scanning 7,400 plots/h, and showed that the heritability of those estimated values was highly repeatable and as high as the heritability of the corresponding ground observations, proposing a practical application in their breeding program.

HTP can also generate new traits that used to be fairly difficult to obtain in the past, such as time series canopy coverage growth patterns over time, providing new approaches to the study of crops. Furthermore, this method may allows to eliminate the need for tedious yield phenotyping after harvest by predicting yield and other desired traits with models based on traits that are more easily obtainable before harvest (Parmley *et al.* 2019), as previously discussed for model-assisted phenotyping.

Despite the recent technological success of HTP, which is promising in the acceleration of crop breeding, few have practically adopted this method or demonstrated its results in plant breeding programs (Awada *et al.* 2018, Deery and Jones 2021). Deery and Jones (2021) emphasized the importance of targeting the needs of breeders rather than pursuing the technologies through the collaboration between phenomics researchers and breeders, while Awada *et al.* (2018) found that how to integrate and utilize enormous amount of data generated by HTP in breeding programs was unclear for plant breeders. In summary, existing HTP technologies are not breeder-oriented but technology-oriented.

Although breeders need an integrated pipeline or tool, most of the HTP technologies that are currently available are segmented, including data management. Thus, it is not easy for breeders to employ them. For example, several UAS applications have been introduced in this paper. Reading the original articles of those applications, although the usage of UAS seems straightforward, in reality it is rather difficult to capture quality images and to process the images before data analysis for phenotyping. As summarized by Guo *et al.* (2021), several complex steps are required to properly acquire and process field images by UAS prior to image processing for phenotyping, making the expected end users hesitate to adopt UAS for their breeding programs.

To solve these issues, the enrichment of easy-to-use phenotyping tools to handle these processes is necessary. EasyIDP (Wang *et al.* 2021a) for intermediate data processing for UAS images, EasyMPE (Tresch *et al.* 2019) for microplot extraction, EasyPCC (Guo *et al.* 2017) for crop segmentation, and EasyDCP (Feldman *et al.* 2021) for 3D phenotyping are good examples. Then, we would need to integrate these tools as a pipeline on a common data exchange platform with standardized application programming interfaces (APIs) and access to genotypic data.

In addition, many of the traits obtained by HTP are given in the estimated values or newly defined ones, and breeders hesitate to replace traditionally obtained values with the estimated values or the values of the newly defined traits. Regarding this issue, discussions of the estimated values for the newly defined traits by HTP are needed among crop scientists, including breeders. For example, there is a need to understand that a widely used index, LAI, is a compromised index that cannot exactly reflect canopy foliage architecture because the light interception of a canopy with the same LAI with different leaf angles should not be the same. Alternatively, NDVI, the most popular vegetation index, has the same background as it was defined when a very limited number of reflectance bands was available. Now that hyperspectral images are becoming available at reasonable prices, we may be able to develop new models to monitor crop physical and physiological status with a much higher dimension and accuracy.

In this review, phenotyping of non-architectural traits, such as nutritious conditions and photosynthetic activities, has not been discussed despite their importance in crop productivity. It is well known that chlorophyll content can be estimated well by using spectral reflectance as commonly used in SPAD measurements, and can be estimated from UAS hyperspectral images (Shu *et al.* 2021). Fu *et al.* (2019) also showed the possibility of estimating the photosynthetic capacity of six tobacco genotypes using a model based on hyperspectral reflectances. Furthermore, the use of light-induced fluorescence transients (LIFT) has been used to estimate photosynthetic activities in open canopies. For example, Keller *et al.* (2019) used LIFT to evaluate photosynthesis in the soybean canopy. A totally different approach was used by Liu *et al.* (2021b), who compared the photosynthetic performances using the virtual canopies of different foliage architectures, as introduced above. Although these technologies look great, they are still far from being practically applied in HTP in the field.

## Author Contribution Statement

S.N. wrote the manuscript.

## References

[B1] Achanta, R., A. Shaji, K. Smith, A. Lucchi, P. Fua and S. Süsstrunk (2012) SLIC superpixels compared to state-of-the-art superpixel methods. IEEE Trans Pattern Anal Mach Intell 34: 2274–2282. 22641706 10.1109/TPAMI.2012.120

[B2] Alkhudaydi, T., D. Reynolds, S. Griffiths, J. Zhou and B. de la Iglesia (2019) An exploration of deep-learning based phenotypic analysis to detect spike regions in field conditions for UK bread wheat. Plant Phenomics 2019: 7368761. 33313535 10.34133/2019/7368761 PMC7706304

[B3] Altas, Z., M.M. Ozguven and Y. Yanar (2018) Determination of sugar beet leaf spot disease level (*Cercospora Beticola Sacc.*) with image processing technique by using drone. Current Investigations in Agriculture and Current Research 5: 621-631.

[B4] Ando, R., Y. Ozasa and W. Guo (2021) Robust surface reconstruction of plant leaves from 3D point clouds. Plant Phenomics 2021: 3184185. 33860276 10.34133/2021/3184185 PMC8038853

[B5] Asaari, M.S.M., S. Mertens, S. Dhondt, D. Inzé, N. Wuyts and P. Scheunders (2019) Analysis of hyperspectral images for detection of drought stress and recovery in maize plants in a high-throughput phenotyping platform. Comput Electron Agric 162: 749–758.

[B6] Atkinson, J.A., M.P. Pound, M.J. Bennett and D.M. Wells (2019) Uncovering the hidden half of plants using new advances in root phenotyping. Curr Opin Biotechnol 55: 1–8. 30031961 10.1016/j.copbio.2018.06.002 PMC6378649

[B7] Atoum, Y., M.J. Afridi, X. Liu, J.M. McGrath and L.E. Hanson (2016) On developing and enhancing plant-level disease rating systems in real fields. Pattern Recognit 53: 287–299.

[B8] Awada, L., P.W.B. Phillips and S.J. Smyth (2018) The adoption of automated phenotyping by plant breeders. Euphytica 214: 148.

[B9] Barbedo, J.G.A. (2019) A review on the use of unmanned aerial vehicles and imaging sensors for monitoring and assessing plant stresses. Drones 3: 40.

[B10] Blancon, J., D. Dutartre, M.H. Tixier, M. Weiss, A. Comar, S. Praud and F. Baret (2019) A High-throughput model-assisted method for phenotyping maize green leaf area index dynamics using unmanned aerial vehicle imagery. Front Plant Sci 10: 685. 31231403 10.3389/fpls.2019.00685 PMC6568052

[B11] Boulent, J., S. Foucher, J. Théau and P.L. St-Charles (2019) Convolutional neural networks for the automatic identification of plant diseases. Front Plant Sci 10: 941. 31396250 10.3389/fpls.2019.00941 PMC6664047

[B12] Bruce, R.W., I. Rajcan and J. Sulik (2021) Classification of soybean pubescence from multispectral aerial imagery. Plant Phenomics 2021: 9806201. 34409302 10.34133/2021/9806201 PMC8363756

[B13] Cai, J., P. Kumar, J. Chopin and S.J. Miklavcic (2018) Land-based crop phenotyping by image analysis: Accurate estimation of canopy height distributions using stereo images. PLoS One 13: e0196671. 29795568 10.1371/journal.pone.0196671 PMC5967702

[B14] Chandra, A.L., S.V. Desai, V.N. Balasubramanian, S. Ninomiya and W. Guo (2020) Active learning with point supervision for cost‑effective panicle detection in cereal crops. Plant Methods 16: 34. 32161624 10.1186/s13007-020-00575-8 PMC7060654

[B15] Coppens, F., N. Wuyts, D. Inze and S. Dhondt (2017) Unlocking the potential of plant phenotyping data through integration and data-driven approaches. Curr Opin Syst Biol 4: 58–63. 32923745 10.1016/j.coisb.2017.07.002 PMC7477990

[B16] Costa, C., U. Schurr, F. Loreto, P. Menesatti and S. Carpentier (2019) Plant phenotyping research trends, a science mapping approach. Front Plant Sci 9: 1933. 30666264 10.3389/fpls.2018.01933 PMC6330294

[B17] Crusiol, L.G.T., M.R. Nanni, R.H. Furlanetto, R.N.R. Sibaldelli, E. Cezar, L.M. Mertz-Henning, A.L. Nepomuceno, N. Neumaier and J.R.B. Farias (2020) UAV-based thermal imaging in the assessment of water status of soybean plants. Int J Remote Sens 41: 3243–3265.

[B18] Csurka, G., C.R. Dance, L. Fan, J. Willamowski and C. Bray (2004) Visual categorization with bags of keypoints. Proceedings European Conference on Computer Vision Workshop on Statistical Learning in Computer Vision 2004: 59–74.

[B19] Ćwiek-Kupczyńska, H., T. Altmann, D. Arend, E. Arnaud, D. Chen, G. Cornut, F. Fiorani, W. Frohmberg, A. Junker, C. Klukas et al. (2016) Measures for interoperability of phenotypic data: Minimum information requirements and formatting. Plant Methods 12: 44. 27843484 10.1186/s13007-016-0144-4 PMC5103589

[B20] Dang, L.M., S. Ibrahim Hassan, I. Suhyeon, A. kumar Sangaiah, I. Mehmood, S. Rho, S. Seo and H. Moon (2020) UAV based wilt detection system via convolutional neural networks. Sustainable Computing: Informatics and Systems 28: 100250.

[B21] David, E., S. Madec, P. Sadeghi-Tehran, H. Aasen, B. Zheng, S. Liu, N. Kirchgessner, G. Ishikawa, K. Nagasawa, M.A. Badhon et al. (2020) Global wheat head detection (GWHD) dataset: A large and diverse dataset of high-resolution RGB-labelled images to develop and benchmark wheat head detection methods. Plant Phenomics 2020: 3521852. 33313551 10.34133/2020/3521852 PMC7706323

[B22] David, E., M. Serouart, D. Smith, S. Madec, K. Velumani, S. Liu, X. Wang, F. Pinto, S. Shafiee, I.S.A. Tahir et al. (2021) Global wheat head detection 2021: An improved dataset for benchmarking wheat dead detection methods. Plant Phenomics 2021: 9846158. 34778804 10.34133/2021/9846158 PMC8548052

[B23] De Castro, A.I., J. Torres-Sánchez, J.M. Peña, F.M. Jiménez-Brenes, O. Csillik and F. López-Granados (2018) An automatic random forest-OBIA algorithm for early weed mapping between and within crop rows using UAV imagery. Remote Sens (Basel) 10: 285.

[B24] DeChant, C., T. Wiesner-Hanks, S. Chen, E.L. Stewart, J. Yosinski, M.A. Gore, R.J. Nelson and H. Lipson (2017) Automated identification of northern leaf blight-infected maize plants from field imagery using deep learning. Phytopathology 107: 1426–1432. 28653579 10.1094/PHYTO-11-16-0417-R

[B25] Deery, D.M., G.J. Rebetzke, J.A. Jimenez-Berni, W.D. Bovill, R.A. James, A.G. Condon, R.T. Furbank, S.C. Chapman and R.A. Fischer (2019) Evaluation of the phenotypic repeatability of canopy temperature in wheat using continuous-terrestrial and airborne measurements. Front Plant Sci 10: 875. 31338102 10.3389/fpls.2019.00875 PMC6629910

[B26] Deery, D.M., G.J. Rebetzke, J.A. Jimenez-Berni, A.G. Condon, D.J. Smith, K.M. Bechaz and W.D. Bovill (2020) Ground-based LiDAR improves phenotypic repeatability of above-ground biomass and crop growth rate in wheat. Plant Phenomics 2020: 8329798. 33313565 10.34133/2020/8329798 PMC7706344

[B27] Deery, D.M. and H.G. Jones (2021) Field phenomics: Will it enable crop improvement? Plant Phenomics 2021: 9871989. 34549194 10.34133/2021/9871989 PMC8433881

[B28] Desai, S.V., V.N. Balasubramanian, T. Fukatsu, S. Ninomiya and W. Guo (2019) Automatic estimation of heading date of paddy rice using deep learning. Plant Methods 15: 76. 31338116 10.1186/s13007-019-0457-1 PMC6626381

[B29] Dobbels, A.A. and A.J. Lorenz (2019) Soybean iron deficiency chlorosis high-throughput phenotyping using an unmanned aircraft system. Plant Methods 15: 97. 31452673 10.1186/s13007-019-0478-9 PMC6700811

[B30] Duan, T., B. Zheng, W. Guo, S. Ninomiya, Y. Guo and S.C. Chapman (2017) Comparison of ground cover estimates from experiment plots in cotton, sorghum and sugarcane based on images and ortho-mosaics captured by UAV. Funct Plant Biol 44: 169–183. 10.1071/FP16123 32480555

[B31] Duarte-Carvajalino, J.M., D.F. Alzate, A.A. Ramirez, J.D. Santa-Sepulveda, A.E. Fajardo-Rojas and M. Soto-Suárez (2018) Evaluating late blight severity in potato crops using unmanned aerial vehicles and machine learning algorithms. Remote Sens (Basel) 10: 1513.

[B32] Fasoula, D.A., I.M. Ioannides and M. Omirou (2020) Phenotyping and plant breeding: Overcoming the barriers. Front Plant Sci 10: 1713. 31998353 10.3389/fpls.2019.01713 PMC6962186

[B33] Feldman, M.J., P.Z. Ellsworth, N. Fahlgren, M.A. Gehan, A.B. Cousins and I. Baxter (2018) Components of water use efficiency have unique genetic signatures in the model C_4_ Grass *Setaria*. Plant Physiol 178: 699–715. 30093527 10.1104/pp.18.00146 PMC6181048

[B34] Feldman, A., H. Wang, Y. Fukano, Y. Kato, S. Ninomiya and W. Guo (2021) EasyDCP: An affordable, high-throughput tool to measure plant phenotypic traits in 3D. Methods Ecol Evol 12: 1679–1686.

[B35] Fernandez-Gallego, J.A., S.C. Kefauver, N.A. Gutiérrez, M.T. Nieto-Taladriz and J.L. Araus (2018) Wheat ear counting in-field conditions: High throughput and low-cost approach using RGB images. Plant Methods 14: 22. 29568319 10.1186/s13007-018-0289-4 PMC5857137

[B36] Fournier, C., B. Andrieu, S. Ljutovac and S. Saint-Jean (2003) ADEL-wheat: A 3D architectural model of wheat development. *In*: Hu, B.-G. and M. Jaeger, M. (eds.) Plant growth modeling and applications, Springer Verlag, Berlin, pp. 54–63.

[B37] Friedli, M., N. Kirchgessner, C. Grieder, F. Liebisch, M. Mannale and A. Walter (2016) Terrestrial 3D laser scanning to track the increase in canopy height of both monocot and dicot crop species under field conditions. Plant Methods 12: 9. 26834822 10.1186/s13007-016-0109-7 PMC4731982

[B38] Fu, P., K. Meacham-Hensold, K. Guan and C.J. Bernacchi (2019) Hyperspectral leaf reflectance as proxy for photosynthetic capacities: An ensemble approach based on multiple machine learning algorithms. Front Plant Sci 10: 730. 31214235 10.3389/fpls.2019.00730 PMC6556518

[B39] Fuentes, A., S. Yoon, S.C. Kim and D.S. Park (2017) A robust deep-learning-based detector for real-time tomato plant diseases and pests recognition. Sensors (Basel) 17: 2022. 10.3390/s17092022 PMC5620500 28869539

[B40] Fuentes, A.F., S. Yoon, J. Lee and D.S. Park (2018) High-performance deep neural network-based tomato plant diseases and pests diagnosis system with refinement filter bank. Front Plant Sci 9: 1162. 30210509 10.3389/fpls.2018.01162 PMC6124392

[B41] Furuta, N., S. Ninomiya, N. Takahashi, H. Ohmori and Y. Ukai (1995) Quantitative evaluation of soybean (Glycine max L. Merr.) leaflet shape by principal component scores based on elliptic Fourier descriptors. Breed Sci 45: 315–320.

[B42] Gage, J.L., E. Richards, N. Lepak, N. Kaczmar, C. Soman, G. Chowdhary, M.A. Gore and E.S. Buckler (2019) In-field whole-plant maize architecture characterized by subcanopy rovers and latent space phenotyping. The Plant Phenome Journal 2: 190011.

[B43] Gamon, J.A., J. Peñuelas and C.B. Field (1992) A narrow-waveband spectral index that tracks diurnal changes in photosynthetic efficiency. Remote Sens Environ 41: 35–44.

[B44] Ghosal, S., D. Blystone, A.K. Singh, B. Ganapathysubramanian, A. Singh and S. Sarkar (2018) An explainable deep machine vision framework for plant stress phenotyping. Proc Natl Acad Sci USA 115: 4613–4618. 29666265 10.1073/pnas.1716999115 PMC5939070

[B45] Ghosal, S., B. Zheng, S.C. Chapman, A.B. Potgieter, D.R. Jordan, X. Wang, A.K. Singh, A. Singh, M. Hirafuji, S. Ninomiya et al. (2019) A weakly supervised deep learning framework for sorghum head detection and counting. Plant Phenomics 2019: 1525874. 33313521 10.34133/2019/1525874 PMC7706102

[B46] Goodfellow, I., J. Pouget-Abadie, M. Mirza, B. Xu, D. Warde-Farley, S. Ozair, A. Courville and Y. Bengio (2014) Generative adversarial nets. *In*: Advances in Neural Information Processing Systems, Proc. 27^th^ Int. Conf. Neural Info. Proc. Sys. Vol. 2 (NIPS’14), MIT Press, Cambridge, MA, USA, pp. 2672–2680.

[B47] Griffiths, M. (2020) A 3D print repository for plant phenomics. Plant Phenomics 2020: 8640215. 33575669 10.34133/2020/8640215 PMC7870102

[B48] Guo, W., U.K. Rage and S. Ninomiya (2013) Illumination invariant segmentation of vegetation for time series wheat images based on decision tree model. Comput Electron Agric 96: 58–66.

[B49] Guo, W., T. Fukatsu and S. Ninomiya (2015) Automated characterization of flowering dynamics in rice using field-acquired time-series RGB images. Plant Methods 11: 7. 25705245 10.1186/s13007-015-0047-9 PMC4336727

[B50] Guo, W., B. Zheng, T. Duan, T. Fukatsu, S. Chapman and S. Ninomiya (2017) EasyPCC: Benchmark datasets and tools for high-throughput measurement of the plant canopy coverage ratio under field conditions. Sensors (Basel) 17: 798. 10.3390/s17040798 PMC5422159 28387746

[B51] Guo, W., B. Zheng, A.B. Potgieter, J. Diot, K. Watanabe, K. Noshita, D.R. Jordan, X. Wang, J. Watson, S. Ninomiya et al. (2018) Aerial imagery analysis—Quantifying appearance and number of sorghum heads for applications in breeding and agronomy. Front Plant Sci 9: 1544. 30405675 10.3389/fpls.2018.01544 PMC6206408

[B52] Guo, W., M.E. Carroll, A. Singh, T.L. Swetnam, N. Merchant, S. Sarkar, A.K. Singh and B. Ganapathysubramanian (2021) UAS-based plant phenotyping for research and breeding applications. Plant Phenomics 2021: 9840192. 34195621 10.34133/2021/9840192 PMC8214361

[B53] Ha, J.G., H. Moon, J.T. Kwak, S.I. Hassan, M. Dang, O.N. Lee and H.Y. Park (2017) Deep convolutional neural network for classifying Fusarium wilt of radish from unmanned aerial vehicles. J Appl Remote Sens 11: 042621.

[B54] Han, L., G. Yang, H. Feng, C. Zhou, H. Yang, B. Xu, Z. Li and X. Yang (2018) Quantitative identification of maize lodging-causing feature factors using unmanned aerial vehicle images and a nomogram computation. Remote Sens (Basel) 10: 1528.

[B55] Han, Y., B.A. Tarakey, S.-J. Hong, S.-Y. Kim, E. Kim, C.-H. Lee and G. Kim (2021) Calibration and image processing of aerial thermal image for UAV application in crop water stress estimation. J Sens 2021: 5537795.

[B56] Häni, N., P. Roy and V. Isler (2020) MinneApple: A benchmark dataset for apple detection and segmentation. IEEE Robot Autom Lett 5: 852–858.

[B57] Hasan, M.M., J.P. Chopin, H. Laga and S.J. Miklavcic (2018) Detection and analysis of wheat spikes using convolutional neural networks. Plant Methods 14: 100. 30459822 10.1186/s13007-018-0366-8 PMC6236889

[B58] Hassan, M.A., M. Yang, A. Rasheed, X. Jin, X. Xia, Y. Xiao and Z. He (2018) Time-series multispectral indices from unmanned aerial vehicle imagery reveal senescence rate in bread wheat. Remote Sens (Basel) 10: 809.

[B59] Hassan, M.A., M. Yang, L. Fu, A. Rasheed, B. Zheng, X. Xia, Y. Xiao and Z. He (2019) Accuracy assessment of plant height using an unmanned aerial vehicle for quantitative genomic analysis in bread wheat. Plant Methods 15: 37. 31011362 10.1186/s13007-019-0419-7 PMC6463666

[B60] Herrero-Huerta, M., A. Bucksch, E. Puttonen and K.M. Rainey (2020) Canopy roughness: A new phenotypic trait to estimate aboveground biomass from unmanned aerial system. Plant Phenomics 2020: 6735967. 33575668 10.34133/2020/6735967 PMC7869937

[B61] Hirafuji, M., H. Yoichi, Y. Miki, T. Kiura, T. Fukatsu, K. Tanaka, K. Matsumoto, N. Hoshi, H. Nesumi, Y. Shibuya et al. (2013) Development of an open Field Server and sensor cloud system. Agricultural Information Research 22: 60–70 (in Japanese with English summary).

[B62] Hoffmann, H., R. Jensen, A. Thomsen, H. Nieto, J. Rasmussen and T. Friborg (2016) Crop water stress maps for an entire growing season from visible and thermal UAV imagery. Biogeosciences 13: 6545–6563.

[B63] Hu, P., S.C. Chapman, X. Wang, A. Potgieter, T. Duan, D. Jordan, Y. Guo and B. Zheng (2018) Estimation of plant height using a high throughput phenotyping platform based on unmanned aerial vehicle and self-calibration: Example for sorghum breeding. Eur J Agron 95: 24–32.

[B64] Hu, P., S.C. Chapman, H. Jin, Y. Guo and B. Zheng (2021) Comparison of modelling strategies to estimate phenotypic values from an unmanned aerial vehicle with spectral and temporal vegetation indexes. Remote Sens (Basel) 13: 2827.

[B65] Huang, H., J. Deng, Y. Lan, A. Yang, X. Deng and L. Zhang (2018) A fully convolutional network for weed mapping of unmanned aerial vehicle (UAV) imagery. PLoS One 13: e0196302. 29698500 10.1371/journal.pone.0196302 PMC5919481

[B66] Isokane, T., F. Okura, A. Ide, Y. Matsushita and Y. Yagi (2018) Probabilistic plant modeling via multi-view image-to-image translation. Proc IEEE Comput Soc Conf Comput Vis Pattern Recognit 2018: 2906–2915.

[B67] Ivushkin, K., H. Bartholomeus, A.K. Bregt, A. Pulatov, M.H.D. Franceschini, H. Kramer, E.N. van Loo, V. Jaramillo Roman and R. Finkers (2019) UAV based soil salinity assessment of cropland. Geoderma 338: 502–512.

[B68] Jay, S., A. Comar, R. Benicio, J. Beauvois, D. Dutartre, G. Daubige, W. Li, J. Labrosse, S. Thomas, N. Henry et al. (2020) Scoring cercospora leaf spot on sugar beet: Comparison of UGV and UAV phenotyping systems. Plant Phenomics 2020: 9452123. 33313567 10.34133/2020/9452123 PMC7706347

[B69] Jiang, Q., S. Fang, Y. Peng, Y. Gong, R. Zhu, X. Wu, Y. Ma, B. Duan and J. Liu (2019) UAV-based biomass estimation for rice-combining spectral, TIN-based structural and meteorological features. Remote Sens (Basel) 11: 890.

[B70] Jiang, Y. and C. Li (2020) Convolutional neural networks for image-based high-throughput plant phenotyping: A review. Plant Phenomics 2020: 4152816. 33313554 10.34133/2020/4152816 PMC7706326

[B71] Jimenez-Berni, J.A., D.M. Deery, P. Rozas-Larraondo, A.G. Condon, G.J. Rebetzke, R.A. James, W.D. Bovill, R.T. Furbank and X.R.R. Sirault (2018) High throughput determination of plant height, ground cover, and above-ground biomass in wheat with LiDAR. Front Plant Sci 9: 237. 29535749 10.3389/fpls.2018.00237 PMC5835033

[B72] Jin, X., S. Madec, D. Dutartre, B. de Solan, A. Comar and F. Baret (2019) High-throughput measurements of stem characteristics to estimate ear density and above-ground biomass. Plant Phenomics 2019: 4820305. 33313528 10.34133/2019/4820305 PMC7706336

[B73] Joalland, S., C. Screpanti, H.V. Varella, M. Reuther, M. Schwind, C. Lang, A. Walter and F. Liebisch (2018) Aerial and ground based sensing of tolerance to beet cyst nematode in sugar beet. Remote Sens (Basel) 10: 787.

[B74] Johansen, K., M.J.L. Morton, Y.M. Malbeteau, B. Aragon, S.K. Al-Mashharawi, M.G. Ziliani, Y. Angel, G.M. Fiene, S.S.C. Negrão, M.A.A. Mousa et al. (2019) Unmanned aerial vehicle-based phenotyping using morphometric and spectral analysis can quantify responses of wild tomato plants to salinity stress. Front Plant Sci 10: 370. 30984222 10.3389/fpls.2019.00370 PMC6449481

[B75] Johnson, J., G. Sharma, S. Srinivasan, S.K. Masakapalli, S. Sharma, J. Sharma and V.K. Dua (2021) Enhanced field-based detection of potato blight in complex backgrounds using deep learning. Plant Phenomics 2021: 9835724. 34104897 10.34133/2021/9835724 PMC8147694

[B76] Kang, H. and C. Chen (2020) Fast implementation of real-time fruit detection in apple orchards using deep learning. Comput Electron Agric 168: 105108.

[B77] Kawamura, K., H. Asai, T. Yasuda, P. Khanthavong, P. Soisouvanh and S. Phongchanmixay (2020) Field phenotyping of plant height in an upland rice field in Laos using low-cost small unmanned aerial vehicles (UAVs). Plant Prod Sci 23: 452–465.

[B78] Keller, B., S. Matsubara, U. Rascher, R. Pieruschka, A. Steier, T. Kraska and O. Muller (2019) Genotype specific photosynthesis × environment interactions captured by automated fluorescence canopy scans over two fluctuating growing seasons. Front Plant Sci 10: 1482. 31998328 10.3389/fpls.2019.01482 PMC6962999

[B79] Khan, Z., J. Chopin, J. Cai, V.-R. Eichi, S. Haefele and S.J. Miklavcic (2018) Quantitative estimation of wheat phenotyping traits using ground and aerial imagery. Remote Sens (Basel) 10: 950.

[B80] Li, B., X. Xu, J. Han, L. Zhang, C. Bian, L. Jin and J. Liu (2019) The estimation of crop emergence in potatoes by UAV RGB imagery. Plant Methods 15: 15. 30792752 10.1186/s13007-019-0399-7 PMC6371461

[B81] Liedtke, J.D., C.H. Hunt, B. George-Jaeggli, K. Laws, J. Watson, A.B. Potgieter, A. Cruickshank and D.R. Jordan (2020) High-throughput phenotyping of dynamic canopy traits associated with stay-green in grain sorghum. Plant Phenomics 2020: 4635153. 33313557 10.34133/2020/4635153 PMC7706314

[B82] Liu, F., P. Hu, B. Zheng, T. Duan, B. Zhu and Y. Guo (2021a) A field-based high-throughput method for acquiring canopy architecture using unmanned aerial vehicle images. Agric For Meteorol 296: 108231.

[B83] Liu, F., Q. Song, J. Zhao, L. Mao, H. Bu, Y. Hu and X.-G. Zhu (2021b) Canopy occupation volume as an indicator of canopy photosynthetic capacity. New Phytol 232: 941–956. 34245568 10.1111/nph.17611

[B84] Liu, J., J. Shang, B. Qian, T. Huffman, Y. Zhang, T. Dong, Q. Jing and T. Martin (2019a) Crop yield estimation using time-series MODIS data and the effects of cropland masks in Ontario, Canada. Remote Sens (Basel) 11: 2419.

[B85] Liu, S., P. Martre, S. Buis, M. Abichou, B. Andrieu and F. Baret (2019b) Estimation of plant and canopy architectural traits using the digital plant phenotyping platform. Plant Physiol 181: 881–890. 31420444 10.1104/pp.19.00554 PMC6836827

[B86] Liu, T., R. Li, X. Jin, J. Ding, X. Zhu, C. Sun and W. Guo (2017) Evaluation of seed emergence uniformity of mechanically sown wheat with UAV RGB imagery. Remote Sens (Basel) 9: 1241.

[B87] Lowe, D.G. (2004) Distinctive image features from scale-invariant keypoints. Int J Comput Vis 60: 91–110.

[B88] Lu, H., Z. Cao, Y. Xiao, B. Zhuang and C. Shen (2017) TasselNet: Counting maize tassels in the wild via local counts regression network. Plant Methods 13: 79. 29118821 10.1186/s13007-017-0224-0 PMC5664836

[B89] Lu, H. and Z. Cao (2020) TasselNetV2+: A fast implementation for high-throughput plant counting from high-resolution RGB imagery. Front Plant Sci 11: 1929. 10.3389/fpls.2020.541960 PMC7750361 33365037

[B90] Lu, N., J. Zhou, Z. Han, D. Li, Q. Cao, X. Yao, Y. Tian, Y. Zhu, W. Cao and T. Cheng (2019) Improved estimation of aboveground biomass in wheat from RGB imagery and point cloud data acquired with a low-cost unmanned aerial vehicle system. Plant Methods 15: 17. 30828356 10.1186/s13007-019-0402-3 PMC6381699

[B91] Lyu, S.X., N. Noguchi, R. Ospina and Y. Kishima (2021) Development of phenotyping system using low altitude UAV imagery and deep learning. International Journal of Agricultural and Biological Engineering 14: 207–215.

[B92] Madec, S., X. Jin, H. Lu, B. De Solan, S. Liu, F. Duyme, E. Heritier and F. Baret (2019) Ear density estimation from high resolution RGB imagery using deep learning technique. Agric For Meteorol 264: 225–234.

[B93] Makanza, R., M. Zaman-Allah, J.E. Cairns, C. Magorokosho, A. Tarekegne, M. Olsen and B.M. Prasanna (2018) High-throughput phenotyping of canopy cover and senescence in maize field trials using aerial digital canopy imaging. Remote Sens (Basel) 10: 330. 33489316 10.3390/rs10020330 PMC7745117

[B94] Miao, C., A. Pages, Z. Xu, E. Rodene, J. Yang and J.C. Schnable (2020) Semantic segmentation of sorghum using hyperspectral data identifies genetic associations. Plant Phenomics 2020: 4216373. 33313555 10.34133/2020/4216373 PMC7706332

[B95] Michez, A., S. Bauwens, Y. Brostaux, M.-P. Hiel, S. Garré, P. Lejeune and B. Dumont (2018) How far can consumer-grade UAV RGB imagery describe crop production? A 3D and multitemporal modeling approach applied to *zea mays*. Remote Sens (Basel) 10: 1798.

[B96] Mirnezami, S.V., S. Srinivasan, Y. Zhou, P.S. Schnable and B. Ganapathysubramanian (2021) Detection of the progression of anthesis in field-grown maize tassels: A case study. Plant Phenomics 2021: 4238701. 33728412 10.34133/2021/4238701 PMC7953991

[B97] Moller, M., V. Alchanatis, Y. Cohen, M. Meron, J. Tsipris, A. Naor, V. Ostrovsky, M. Sprintsin and S. Cohen (2007) Use of thermal and visible imagery for estimating crop water status of irrigated grapevine. Exp Bot 58: 827–838. 10.1093/jxb/erl115 16968884

[B98] Mu, Y., T.-S. Chen, S. Ninomiya and W. Guo (2020) Intact detection of highly occluded immature tomatoes on plants using deep learning techniques. Sensors (Basel) 20: 2984. 10.3390/s20102984 PMC7288109 32466108

[B99] Parmley, K., K. Nagasubramanian, S. Sarkar, B. Ganapathysubramanian and A.K. Singh (2019) Development of optimized phenomic predictors for efficient plant breeding decisions using phenomic-assisted selection in soybean. Plant Phenomics 2019: 5809404. 33313530 10.34133/2019/5809404 PMC7706298

[B100] Patrick, A., S. Pelham, A. Culbreath, C.C. Holbrook, I.J. De Godoy and C. Li (2017) High throughput phenotyping of tomato spot wilt disease in peanuts using unmanned aerial systems and multispectral imaging. IEEE Instrum Meas Mag 20: 4–12.

[B101] Paulus, S., J. Dupuis, S. Riedel and H. Kuhlmann (2014) Automated analysis of barley organs using 3D laser scanning: An approach for high throughput phenotyping. Sensors (Basel) 14: 12670–12686. 25029283 10.3390/s140712670 PMC4168454

[B102] Peng, X., W. Han, J. Ao and Y. Wang (2021) Assimilation of LAI derived from UAV multispectral data into the SAFY model to estimate maize yield. Remote Sens (Basel) 13: 1094.

[B103] Perich, G., A. Hund, J. Anderegg, L. Roth, M.P. Boer, A. Walter, F. Liebisch and H. Aasen (2020) Assessment of multi-image unmanned aerial vehicle based high-throughput field phenotyping of canopy temperature. Front Plant Sci 11: 150. 32158459 10.3389/fpls.2020.00150 PMC7052280

[B104] Phan, A.T.T., K. Takahashi, A. Rikimaru and Y. Higuchi (2016) Method for estimating rice plant height without ground surface detection using laser scanner measurement. J Apple Remote Sens 10: 046018.

[B105] Pommier, C., C. Michotey, G. Cornut, P. Roumet, E. Duchêne, R. Flores, A. Lebreton, M. Alaux, S. Durand, E. Kimmel et al. (2019) Applying FAIR principles to plant phenotypic data management in GnpIS. Plant Phenomics 2019: 1671403. 33313522 10.34133/2019/1671403 PMC7718628

[B106] Rebetzke, G., R. Fischer, D. Deery, J. Jimenez-Berni and D. Smith (2019) Review: High-throughput phenotyping to enhance the use of crop genetic resources. Plant Sci 282: 40–48. 31003610 10.1016/j.plantsci.2018.06.017

[B107] Riera, L.G., M.E. Carroll, Z. Zhang, J.M. Shook, S. Ghosal, T. Gao, A. Singh, S. Bhattacharya, B. Ganapathysubramanian, A.K. Singh et al. (2021) Deep multiview image fusion for soybean yield estimation in breeding applications. Plant Phenomics 2021: 9846470. 34250507 10.34133/2021/9846470 PMC8240512

[B108] Roitsch, T., L. Cabrera-Bosquet, A. Fournier, K. Ghamkhar, J. Jiménez-Berni, F. Pinto and E.S. Ober (2019) Review: New sensors and data-driven approaches—A path to next generation phenomics. Plant Sci 282: 2–10. 31003608 10.1016/j.plantsci.2019.01.011 PMC6483971

[B109] Romero, A.P., A. Alarcón, R.I. Valbuena and C.H. Galeano (2017) Physiological assessment of water stress in potato using spectral information. Front Plant Sci 8: 1608. 28979277 10.3389/fpls.2017.01608 PMC5611683

[B110] Romero, M., Y. Luo, B. Su and S. Fuentes (2018) Vineyard water status estimation using multispectral imagery from an UAV platform and machine learning algorithms for irrigation scheduling management. Comput Electron Agric 147: 109–117.

[B111] Roth, L., M. Camenzind, H. Aasen, L. Kronenberg, C. Barendregt, K.-H. Camp, A. Walter, N. Kirchgessner and A. Hund (2020) Repeated multiview imaging for estimating seedling tiller counts of wheat genotypes using drones. Plant Phenomics 2020: 3729715. 33313553 10.34133/2020/3729715 PMC7706335

[B112] Russakovsky, O., J. Deng, H. Su, J. Krause, S. Satheesh, S. Ma, Z. Huang, A. Karpathy, A. Khosla, M. Bernstein et al. (2015) ImageNet Large scale visual recognition challenge. Int J Comput Vis 115: 211–252.

[B113] Sa, I., Z. Ge, F. Dayoub, B. Upcroft, T. Perez and C. McCool (2016) DeepFruits: A fruit detection system using deep neural networks. Sensors (Basel) 16: 1222. 10.3390/s16081222 PMC5017387 27527168

[B114] Sadeghi-Tehran, P., N. Virlet, E.M. Ampe, P. Reyns and M.J. Hawkesford (2019) *DeepCount*: In-field automatic quantification of wheat spikes using simple linear iterative clustering and deep convolutional neural networks. Front Plant Sci 10: 1176. 31616456 10.3389/fpls.2019.01176 PMC6775245

[B115] Sagan, V., M. Maimaitijiang, P. Sidike, K. Eblimit, K.T. Peterson, S. Hartling, F. Esposito, K. Khanal, M. Newcomb, D. Pauli et al. (2019) UAV-based high resolution thermal imaging for vegetation monitoring, and plant phenotyping using ICI 8640 P, FLIR Vue Pro R 640, and thermomap cameras. Remote Sens (Basel) 11: 330.

[B116] Schirrmann, M., N. Landwehr, A. Giebel, A. Garz and K.-H. Dammer (2021) Early detection of stripe rust in winter wheat using deep residual neural networks. Front Plant Sci 12: 475. 10.3389/fpls.2021.469689 PMC8042394 33859655

[B117] Seguin, B., J.P. Lagouarde and M. Savane (1991) The assessment of regional crop water conditions from meteorological satellite thermal infrared data. Remote Sens Environ 35: 141–148.

[B118] Selby, P., R. Abbeloos, J.E. Backlund, M. Basterrechea Salido, G. Bauchet, O.E. Benites-Alfaro, C. Birkett, V.C. Calaminos, P. Carceller, G. Cornut et al. (2019) BrAPI—an application programming interface for plant breeding applications. Bioinformatics 35: 4147–4155. 30903186 10.1093/bioinformatics/btz190 PMC6792114

[B119] Shete, S., S. Srinivasan and T.A. Gonsalves (2020) TasselGAN: An Application of the generative adversarial model for creating field-based maize tassel data. Plant Phenomics 2020: 8309605. 33313564 10.34133/2020/8309605 PMC7706316

[B120] Shrestha, R., L. Matteis, M. Skofic, A. Portugal, G. McLaren, G. Hyman and E. Arnaud (2012) Bridging the phenotypic and genetic data useful for integrated breeding through a data annotation using the crop ontology developed by the crop communities of practice. Front Physiol 3: 326. 22934074 10.3389/fphys.2012.00326 PMC3429094

[B121] Shu, M., M. Shen, J. Zuo, P. Yin, M. Wang, Z. Xie, J. Tang, R. Wang, B. Li, X. Yang et al. (2021) The application of UAV-based hyperspectral imaging to estimate crop traits in maize inbred lines. Plant Phenomics 2021: 9890745. 33889850 10.34133/2021/9890745 PMC8054988

[B122] Singh, A., B. Ganapathysubramanian, A.K. Singh and S. Sarkar (2016) Machine learning for high-throughput stress phenotyping in plants. Trend Plant Sci 21: 110–124. 10.1016/j.tplants.2015.10.015 26651918

[B123] Singh, A.K., B. Ganapathysubramanian, S. Sarkar and A. Singh (2018) Deep learning for plant stress phenotyping: Trends and future perspectives. Trend Plant Sci 23: 883–898. 10.1016/j.tplants.2018.07.004 30104148

[B124] Singh, A., S. Jones, B. Ganapathysubramanian, S. Sarkar, D. Mueller, K. Sandhu and K. Nagasubramanian (2021) Challenges and opportunities in machine-augmented plant stress phenotyping. Trends Plant Sci 26: 53–69. 32830044 10.1016/j.tplants.2020.07.010

[B125] Singh, D., X. Wang, U. Kumar, L. Gao, M. Noor, M. Imtiaz, R.P. Singh and J. Poland (2019) High-throughput phenotyping enabled genetic dissection of crop lodging in wheat. Front Plant Sci 10: 394. 31019521 10.3389/fpls.2019.00394 PMC6459080

[B126] Singh, V., A. Rana, M. Bishop, A.M. Filippi, D. Cope, N. Rajan and M. Bagavathiannan (2020) Chapter Three—Unmanned aircraft systems for precision weed detection and management: Prospects and challenges. Advances in Agronomy 159: 93–134.

[B127] Skovsen, S.K., M.S. Laursen, R.K. Kristensen, J. Rasmussen, M. Dyrmann, J. Eriksen, R. Gislum, R.N. Jørgensen and H. Karstoft (2021) Robust species distribution mapping of crop mixtures using color images and convolutional neural networks. Sensors (Basel) 21: 175. 10.3390/s21010175 PMC7794678 33383904

[B128] Srivastava, S., S. Bhugra, B. Lall and S. Chaudhury (2017) Drought stress classification using 3D plant models. IEEE Int Conf Comput Vis Workshops, pp. 2046–2054.

[B129] Su, J., C. Liu, M. Coombes, X. Hu, C. Wang, X. Xu, Q. Li, L. Guo and W.-H. Chen (2018) Wheat yellow rust monitoring by learning from multispectral UAV aerial imagery. Comput Electron Agric 155: 157–166.

[B130] Sugiura, R., S. Tsuda, S. Tamiya, A. Itoh, K. Nishiwaki, N. Murakami, Y. Shibuya, M. Hirafuji and S. Nuske (2016) Field phenotyping system for the assessment of potato late blight resistance using RGB imagery from an unmanned aerial vehicle. Biosyst Eng 148: 1–10.

[B131] Sun, Q., L. Sun, M. Shu, X. Gu, G. Yang and L. Zhou (2019) Monitoring maize lodging grades via unmanned aerial vehicle multispectral image. Plant Phenomics 2019: 5704154. 33313529 10.34133/2019/5704154 PMC7706340

[B132] Sun, S., C. Li, A.H. Paterson, Y. Jiang, R. Xu, J.S. Robertson, J.L. Snider and P.W. Chee (2018) In-field high throughput phenotyping and cotton plant growth analysis using LiDAR. Front Plant Sci 9: 16. 29403522 10.3389/fpls.2018.00016 PMC5786533

[B133] Tanger, P., S. Klassen, J.P. Mojica, J.T. Lovell, B.T. Moyers, M. Baraoidan, M.E.B. Naredo, K.L. McNally, J. Poland, D.R. Bush et al. (2017) Field-based high throughput phenotyping rapidly identifies genomic regions controlling yield components in rice. Sci Rep 7: 42839. 28220807 10.1038/srep42839 PMC5318881

[B134] Teramoto, S. and Y. Uga (2022) Improving the efficiency of plant root system phenotyping through digitization and automation. Breed Sci 72: 48–55. 10.1270/jsbbs.21053 PMC8987843 36045896

[B135] Tetila, E.C., B.B. Machado, N.A. Belete, D.A. Guimaraes and H. Pistori (2017) Identification of soybean foliar diseases using unmanned aerial vehicle images. IEEE Geosci Remote Sens Lett 14: 2190–2194.

[B136] Thomas, S., J. Behmann, A. Steier, T. Kraska, O. Muller, U. Rascher and A.-K. Mahlein (2018) Quantitative assessment of disease severity and rating of barley cultivars based on hyperspectral imaging in a non-invasive, automated phenotyping platform. Plant Methods 14: 45. 29930695 10.1186/s13007-018-0313-8 PMC5994119

[B137] Thorp, K.R., A.L. Thompson, S.J. Harders, A.N. French and R.W. Ward (2018) High-throughput phenotyping of crop water use efficiency via multispectral drone imagery and a daily soil water balance model. Remote Sens (Basel) 10: 1682.

[B138] Tilly, N., D. Hoffmeister, Q. Cao, S. Huang, V. Lenz-Wiedemann, Y. Miao and G. Bareth (2014) Multitemporal crop surface models: Accurate plant height measurement and biomass estimation with terrestrial laser scanning in paddy rice. J Apple Remote Sens 8: 1–23.

[B139] Toda, Y. and F. Okura (2019) How convolutional neural networks diagnose plant disease. Plant Phenomics 2019: 9237136. 33313540 10.34133/2019/9237136 PMC7706313

[B140] Toda, Y., F. Okura, J. Ito, S. Okada, T. Kinoshita, H. Tsuji and D. Saisho (2020) Training instance segmentation neural network with synthetic datasets for crop seed phenotyping. Commun Biol 3: 173. 32296118 10.1038/s42003-020-0905-5 PMC7160130

[B141] Torres-Sánchez, J., F.J. Mesas-Carrascosa, F.M. Jiménez-Brenes, A.I. de Castro and F. López-Granados (2021) Early detection of broad-leaved and grass weeds in wide row crops using artificial neural networks and UAV imagery. Agronomy 11: 749.

[B142] Tresch, L., Y. Mu, A. Itoh, A. Kaga, K. Taguchi, M. Hirafuji, S. Ninomiya and W. Guo (2019) Easy MPE: Extraction of quality microplot images for UAV-based high-throughput field phenotyping. Plant Phenomics 2019: 2591849. 33313523 10.34133/2019/2591849 PMC7706339

[B143] Ubbens, J., M. Cieslak, P. Prusinkiewicz, I. Parkin, J. Ebersbach and I. Stavness (2020) Latent space phenotyping: Automatic image-based phenotyping for treatment studies. Plant Phenomics 2020: 5801869. 33313558 10.34133/2020/5801869 PMC7706325

[B144] Uga, Y. (2021) Challenges to design-oriented breeding of root system architecture adapted to climate change. Breed Sci 71: 3–12. 33762871 10.1270/jsbbs.20118 PMC7973499

[B145] Velumani, K., R. Lopez-Lozano, S. Madec, W. Guo, J. Gillet, A. Comar and F. Baret (2021) Estimates of maize plant density from UAV RGB images using faster-RCNN detection model: Impact of the spatial resolution. Plant Phenomics 2021: 9824843. 34549193 10.34133/2021/9824843 PMC8404552

[B146] Wakamori, K. and H. Mineno (2019) Optical flow-based analysis of the relationships between leaf wilting and stem diameter variations in tomato plants. Plant Phenomics 2019: 9136298. 33313538 10.34133/2019/9136298 PMC7706306

[B147] Walter, J., J. Edwards, J. Cai, G. McDonald, S.J. Miklavcic and H. Kuchel (2019a) High-throughput field imaging and basic image analysis in a wheat breeding programme. Front Plant Sci 10: 449. 31105715 10.3389/fpls.2019.00449 PMC6492763

[B148] Walter, J.D.C., J. Edwards, G. McDonald and H. Kuchel (2019b) Estimating biomass and canopy height with LiDAR for field crop breeding. Front Plant Sci 10: 1145. 31611889 10.3389/fpls.2019.01145 PMC6775483

[B149] Wang, H., Y. Duan, Y. Shi, Y. Kato, S. Ninomiya and W. Guo (2021a) EasyIDP: A Python package for intermediate data processing in UAV-based plant phenotyping. Remote Sens (Bazel) 13: 2622.

[B150] Wang, H., S. Lyu and Y. Ren (2021b) Paddy rice imagery dataset for panicle segmentation. Agronomy 11: 1542.

[B151] Wang, J., B. Wu, M.V. Kohnen, D. Lin, C. Yang, X. Wang, A. Qiang, W. Liu, J. Kang, H. Li et al. (2021c) Classification of rice yield using UAV-based hyperspectral imagery and lodging feature. Plant Phenomics 2021: 9765952. 33851136 10.34133/2021/9765952 PMC8028843

[B152] Wang, X., R. Zhang, W. Song, L. Han, X. Liu, X. Sun, M. Luo, K. Chen, Y. Zhang, H. Yang et al. (2019) Dynamic plant height QTL revealed in maize through remote sensing phenotyping using a high-throughput unmanned aerial vehicle (UAV). Sci Rep 9: 3458. 30837510 10.1038/s41598-019-39448-z PMC6401315

[B153] Watanabe, K., W. Guo, K. Arai, H. Takanashi, H. Kajiya-Kanegae, M. Kobayashi, K. Yano, T. Tokunaga, T. Fujiwara, N. Tsutsumi et al. (2017) High-throughput phenotyping of sorghum plant height using an unmanned aerial vehicle and its application to genomic prediction modeling. Front Plant Sci 8: 421. 28400784 10.3389/fpls.2017.00421 PMC5368247

[B154] Watt, M., F. Fiorani, B. Usadel, U. Rascher, O. Muller and U. Schurr (2020) Phenotyping: New windows into the plant for breeders. Annu Rev Plant Biol 71: 689–712. 32097567 10.1146/annurev-arplant-042916-041124

[B155] Wiesner-Hanks, T., H. Wu, E. Stewart, C. DeChant, N. Kaczmar, H. Lipson, M.A. Gore and R.J. Nelson (2019) Millimeter-level plant disease detection from aerial photographs *via* deep learning and crowdsourced data. Front Plant Sci 10: 1550. 31921228 10.3389/fpls.2019.01550 PMC6927297

[B156] Wilke, N., B. Siegmann, L. Klingbeil, A. Burkart, T. Kraska, O. Muller, A. van Doorn, S. Heinemann and U. Rascher (2019) Quantifying lodging percentage and lodging severity using a UAV-based canopy height model combined with an objective threshold approach. Remote Sens (Basel) 11: 515.

[B157] Wilkinson, M.D., M. Dumontier, I.J. Aalbersberg, G. Appleton, M. Axton, A. Baak, N. Blomberg, J.-W. Boiten, L.B. da Silva Santos, P.E. Bourne et al. (2016) The FAIR Guiding Principles for scientific data management and stewardship. Sci Data 3: 160018. 26978244 10.1038/sdata.2016.18 PMC4792175

[B158] Woebbecke, D.M., G.E. Meyer, K. Von Bargen and D.A. Mortensen (1995) Color indices for weed identification under various soil residue and lighting conditions. Biol Eng Trans 38: 259–269.

[B159] Wu, J., G. Yang, X. Yang, B. Xu, L. Han and Y. Zhu (2019) Automatic counting of *in situ* rice seedlings from UAV images based on a deep fully convolutional neural network. Remote Sens (Basel) 11: 691.

[B160] Xiao, S., H. Chai, K. Shao, M. Shen, Q. Wang, R. Wang, Y. Sui and Y. Ma (2020) Image-based dynamic quantification of aboveground structure of sugar beet in field. Remote Sens (Basel) 12: 269.

[B161] Xiong, H., Z. Cao, H. Lu, S. Madec, L. Liu and C. Shen (2019) TasselNetv2: In-field counting of wheat spikes with context-augmented local regression networks. Plant Methods 15: 150. 31857821 10.1186/s13007-019-0537-2 PMC6905110

[B162] Xiong, X., L. Duan, L. Liu, H. Tu, P. Yang, D. Wu, G. Chen, L. Xiong, W. Yang and Q. Liu (2017) Panicle-SEG: A robust image segmentation method for rice panicles in the field based on deep learning and superpixel optimization. Plant Methods 13: 104. 29209408 10.1186/s13007-017-0254-7 PMC5704426

[B163] Yao, X., N. Wang, Y. Liu, T. Cheng, Y. Tian, Q. Chen and Y. Zhu (2017) Estimation of wheat LAI at middle to high levels using unmanned aerial vehicle narrowband multispectral imagery. Remote Sens (Basel) 9: 1304.

[B164] Yates, S., A. Mikaberidze, S.G. Krattinger, M. Abrouk, A. Hund, K. Yu, B. Studer, S. Fouche, L. Meile, D. Pereira et al. (2019) Precision phenotyping reveals novel loci for quantitative resistance to septoria tritici blotch. Plant Phenomics 2019: 3285904. 33313526 10.34133/2019/3285904 PMC7706307

[B165] Yeom, J., J. Jung, A. Chang, M. Maeda and J. Landivar (2018) Automated open cotton boll detection for yield estimation using unmanned aircraft vehicle (UAV) data. Remote Sens (Basel) 10: 1895.

[B166] Yoshioka, Y., H. Iwata, R. Ohsawa and S. Ninomiya (2004) Quantitative evaluation of flower colour pattern by image analysis and principal component analysis in *Primula sieboldii* E. Morren. Eupytica 139: 179–186.

[B167] Yuan, H., R.S. Bennett, N. Wang and K.D. Chamberlin (2019) Development of a peanut canopy measurement system using a ground-based LiDAR sensor. Front Plant Sci 10: 203. 30873193 10.3389/fpls.2019.00203 PMC6403138

[B168] Yue, J., H. Feng, G. Yang and Z. Li (2018a) A Comparison of regression techniques for estimation of above-ground winter wheat biomass using near-surface spectroscopy. Remote Sens (Basel) 10: 66.

[B169] Yue, J., H. Feng, X. Jin, H. Yuan, Z. Li, C. Zhou, G. Yang and Q. Tian (2018b) A comparison of crop parameters estimation using images from UAV-mounted snapshot hyperspectral sensor and high-definition digital camera. Remote Sens (Basel) 10: 1138.

[B170] Zhang, D., X. Zhou, J. Zhang, Y. Lan, C. Xu and D. Liang (2018) Detection of rice sheath blight using an unmanned aerial system with high-resolution color and multispectral imaging. PLoS One 13: e0187470. 29746473 10.1371/journal.pone.0187470 PMC5945033

[B171] Zhang, L., C.L. Guo, L.Y. Zhao, Y. Zhu, W.X. Cao, Y.C. Tian, T. Cheng and X. Wang (2016) Estimating wheat yield by integrating the WheatGrow and PROSAIL models. Field Crops Res 192: 55–66.

[B172] Zhang, L., Y. Niu, H. Zhang, W. Han, G. Li, J. Tang and X. Peng (2019) Maize canopy temperature extracted from UAV thermal and RGB imagery and its application in water stress monitoring. Front Plant Sci 10: 1270. 31649715 10.3389/fpls.2019.01270 PMC6794609

[B173] Zhang, W., K. Chen, J. Wang, Y. Shi and W. Guo (2021) Easy domain adaptation method for filling the species gap in deep learning-based fruit detection. Hortic Res 8: 119. 34059636 10.1038/s41438-021-00553-8 PMC8167097

[B174] Zhao, C., Y. Zhang, J. Du, X. Guo, W. Wen, S. Gu, J. Wang and J. Fan (2019) Crop phenomics: Current status and perspectives. Front Plant Sci 10: 714. 31214228 10.3389/fpls.2019.00714 PMC6557228

[B175] Zhao, J., X. Zhang, J. Yan, X. Qiu, X. Yao, Y. Tian, Y. Zhu and W. Cao (2021a) A wheat spike detection method in UAV images based on improved YOLOv5. Remote Sens (Basel) 13: 3095.

[B176] Zhao, L., W. Guo, J. Wang, H. Wang, Y. Duan, C. Wang, W. Wu and Y. Shi (2021b) An efficient method for estimating wheat heading dates using UAV images. Remote Sens (Basel) 13: 3067.

[B177] Zhou, C., H. Ye, J. Hu, X. Shi, A. Hua, J. Yue, Z. Xu and G. Yang (2019) Automated counting of rice panicle by applying deep learning model to images from unmanned aerial vehicle platform. Sensors (Basel) 19: 3106. 10.3390/s19143106 PMC6679257 31337086

[B178] Zhou, J., H. Mou, J. Zhou, M.L. Ali, H. Ye, P. Chen and H.T. Nguyen (2021) Qualification of soybean responses to flooding stress using UAV-based imagery and deep learning. Plant Phenomics 2021: 9892570. 34286285 10.34133/2021/9892570 PMC8261669

[B179] Zhu, J.Y., T. Park, P. Isola and A.A. Efros (2017) Unpaired image-to-image translation using cycle-consistent adversarial networks. IEEE Int Conf Comput Vis Workshops 2017: 2223–2232.

[B180] Ziliani, M.G., S.D. Parkes, I. Hoteit and M.F. McCabe (2018) Intra-season crop height variability at commercial farm scales using a fixed-wing UAV. Remote Sens (Basel) 10: 2007.

